# Insertions and Duplications in the Polyproline Region of the Hepatitis E Virus

**DOI:** 10.3389/fmicb.2020.00001

**Published:** 2020-01-31

**Authors:** Sébastien Lhomme, Florence Nicot, Nicolas Jeanne, Chloé Dimeglio, Alain Roulet, Caroline Lefebvre, Romain Carcenac, Maxime Manno, Martine Dubois, Jean-Marie Peron, Laurent Alric, Nassim Kamar, Florence Abravanel, Jacques Izopet

**Affiliations:** ^1^Laboratoire de Virologie, Centre National de Référence du virus de l’hépatite E, Hôpital Purpan, CHU de Toulouse, Toulouse, France; ^2^INSERM, U1043, Toulouse, France; ^3^Université Toulouse III-Paul Sabatier, Toulouse, France; ^4^Plateforme Génomique, Centre INRA Occitanie-Toulouse, Castanet-Tolosan, France; ^5^Service de Gastroentérologie, Hôpital Purpan, CHU de Toulouse, Toulouse, France; ^6^Service de médecine interne, Hôpital Purpan, CHU de Toulouse, Toulouse, France; ^7^Service de néphrologie, Dialyse et Transplantation d’Organe, Hôpital Rangueil, CHU de Toulouse, Toulouse, France

**Keywords:** hepatitis E virus, polyproline region, genomic rearrangement, virus-host recombinant variants, virus-virus recombinant variants

## Abstract

Recombinant strains of hepatitis E virus (HEV) with insertions of human genomic fragments or HEV sequence duplications in the sequence encoding the polyproline region (PPR) were previously described in chronically infected patients. Such genomic rearrangements confer a replicative advantage *in vitro* but little is known about their frequency, location, or origin. As the sequences of only a few virus genomes are available, we analyzed the complete genomes of 114 HEV genotype 3 strains from immunocompromised (*n* = 85) and immunocompetent (*n* = 29) patients using the single molecular real-time sequencing method to determine the frequency, location, and origin of inserted genomic fragments, plus the proportions of variants with genomic rearrangements in each virus quasispecies. We also examined the amino acid compositions and post-translational modifications conferred by these rearrangements by comparing them to sequences without human gene insertions or HEV gene duplications. We found genomic rearrangements in 7/114 (6.1%) complete genome sequences (4 HEV-3f, 1 HEV-3e, 1 HEV-3 h, and 1 HEV-3chi-new), all from immunocompromised patients, and 3/7 were found at the acute phase of infection. Six of the seven patients harbored virus-host recombinant variants, including one patient with two different recombinant variants. We also detected three recombinant variants with genome duplications of the PPR or PPR + X domains in a single patient. All the genomic rearrangements (seven human fragment insertions of varying origins and three HEV genome duplications) occurred in the PPR. The sequences with genomic rearrangements had specific characteristics: increased net load (*p* < 0.001) and more ubiquitination (*p* < 0.001), phosphorylation (*p* < 0.001), and acetylation (*p* < 0.001) sites. The human fragment insertions and HEV genome duplications had slightly different characteristics. We believe this is the first description of HEV strains with genomic rearrangements in patients at the acute phase of infection; perhaps these strains are directly transmitted. Clearly, genomic rearrangements produce a greater net load with duplications and insertions having different features. Further studies are needed to clarify the mechanisms by which such modifications influence HEV replication.

## Introduction

The hepatitis E virus (HEV) is a significant human pathogen causing viral hepatitis worldwide. HEV is a member of the *Hepeviridae* family. The genus *Orthohepevirus* includes mammalian and avian strains while the genus *Piscihepevirus* infects Cutthroat Trout (Smith and Simmonds, 2018). The strains infecting humans belong to the *Orthohepevirus A* species. The most prevalent genotype in the industrialized countries, at least in Europe and America, is HEV genotype 3 (HEV-3); it has three major clades: HEV-3abjchi, HEV-3efg, and HEV-3ra (Oliveira-Filho et al., 2013; Smith et al., 2016). The first two clades are mainly found in humans, pigs, wild boar, and deer, and the third in humans and rabbits (Izopet et al., 2012; Abravanel et al., 2017). HEV-3 infections are frequently asymptomatic but they can result in severe acute hepatitis in patients with chronic liver disease (Kamar et al., 2017). HEV-3 can also lead to chronic infection, defined by replication that persists for over 3 months, in immunocompromised patients, including solid organ transplant recipients, patients with hematological disease, and those with an HIV infection. Patients with either acute or chronic hepatitis can suffer from extra-hepatic manifestations (Kamar et al., 2017).

The HEV genome is a single stranded positive-sense RNA about 7.2 kb long that has three open reading frames (ORFs). ORF2 encodes the capsid protein, ORF3 encodes a phosphoprotein involved in virus egress (Kenney and Meng, 2019), and ORF1 encodes a non-structural protein. This protein has several regions: a methyltransferase, a Y domain, a papain-like domain, a polyproline region (PPR), an X domain, a helicase, and an RNA-dependent RNA polymerase (RdRp). The length of the PPR can vary from 189 to 315 nt, depending on the HEV clade and/or subtype. The main PPR length of HEV-3f strains may be 228 nt (HEV-3f-short) or 315 nt (HEV-3f-long) (Lhomme et al., 2014; Nicot et al., 2018). The PPR may be involved in virus adaptation (Shukla et al., 2011; Purdy et al., 2012). The HEV strains infecting chronically HEV-infected patients can contain fragments of human genes, including the S17 ribosomal gene (RPS17), RPS19, human tyrosine aminotransferase gene (TAT), inter-α-trypsin inhibitor gene (ITI) (Shukla et al., 2011; Nguyen et al., 2012; Lhomme et al., 2014), and duplications of the PPR or PPR + RdRp (Johne et al., 2014; Lhomme et al., 2014). This suggests that a prolonged HEV infection favors genomic rearrangements in the PPR but the contribution of an impaired immune response to these recombinant events is not clear. Several *in vitro* studies have shown that a human fragment (RPS17, RPS19, ITI) inserted in the PPR confers a replicative advantage over variants with no human fragments (Shukla et al., 2011; Nguyen et al., 2012; Lhomme et al., 2014), while the duplication of part of the HEV genome (PPR + RdRp) permits HEV adaptation in A549 cell line (Johne et al., 2014). However, the mechanisms that promote virus growth and/or adaptation are largely unknown because of a lack of data.

This study used single molecular real-time (SMRT) sequencing to identify new recombinant HEV genomes, and determine their frequency, location, and origin. We estimated the proportions of variants with genomic rearrangements in each virus quasispecies and identified the features (amino acid composition and post-translational modifications) conferred by the genomic rearrangement and whether human insertions and duplications resulted in different features.

## Materials and Methods

### Patients and Samples

We used stored plasma samples (stored at −80°C) from HEV-infected patients consecutively tested for HEV RNA between 2005 and 2016 in the laboratory of Virology at Toulouse University Hospital, National Reference Center for HEV, where French laboratories can send samples for diagnosis and genotyping. These patients were acutely (HEV replication persisting for less than 3 months) or chronically HEV-infected (HEV replication persisting for more than 3 months). We selected 114 samples containing high HEV virus loads (>100,000 copies/ml) for PacBio SMRT sequencing. The HEV RNA concentrations were determined using a validated real-time polymerase chain reaction (Abravanel et al., 2012). This non-interventional study was supported by Toulouse University Hospital Center. The samples used were part of a collection identified by the French authorities (AC-2015-2518).

The positive control for PacBio SMRT sequencing was the strain VHP6 (passage 6 of TLS-09/M48 virus from the feces of an HEV-infected patient) cultured on HepG2/C3A cells (Lhomme et al., 2014), with two different human genome insertions in the PPR: a fragment of the L-arginine/glycine amidinotransferase (GATM) gene and a fragment of phosphatidylethanolamine binding protein 1 (PEBP1), each variant representing 50% of the quasispecies. Both were characterized by shot-gun deep sequencing (454 GS Junior system). Briefly, six overlapping amplicons were generated. For the library preparation, amplicons were nebulized according to 454 shotgun protocol (Roche/454-Life sciences) and the purified fragmented DNA was further processed according to 454 GS Junior Library construction protocol (Roche/454-Life sciences). The sequencing run was carried out on a Genome Sequencer Junior according to manufacturer instructions (Roche-454 LifeSciences). Data analysis was done with GS *de Novo* Assembler and GS Reference Mapper software.

### Single Molecular Real-Time Sequencing of the Complete Hepatitis E Virus Genome

Full length sequences of the HEV genomic RNA were generated as previously described (Nicot et al., 2018). Briefly, two long amplicons (4,500 and 4,200 bp) with an overlap of around 1,450 bp were amplified and then sequenced using P6-C4 chemistry on the PacBio RSII sequencer [at the Toulouse genomic platform^1^] to obtain the entire 7,250 bp HEV genome. The raw PacBio sequences were processed as previously described by a bioinformatics pipeline and manual processing to reconstruct the individual consensus sequences of each complete HEV genome. Several consensus sequences were sometimes obtained for a single strain indicating the possible presence of different variants in the virus quasispecies. The HEV genotype was determined by analyzing the complete genome sequence by maximum likelihood phylogenetic analysis (Nicot et al., 2018); all the samples contained HEV genotype 3 (HEV-3). The proportion of each variant was estimated using the count related to each consensus read after the processing on Long Amplicon Analysis v2.0.

The detection of recombinant viruses by SMRT sequencing was validated using the positive control VHP6 characterized by shot-gun deep sequencing. Two variants with inserted fragments were detected using SMRT sequencing: one harboring a fragment of GATM and the other harboring a fragment of PEBP1 (Supplementary Figure S1). SMRT sequencing also enabled us to estimate the proportion of each: 50% for VHP6-GATM and 50% for VHP6-PEBP1. Thus, the use of SMRT is appropriate to detect inserted fragments and to determine their proportions.

### Complete Genome Annotation

Each complete genome was annotated to determine the three open reading frames and the length of the domains in ORF1 (methyltransferase, Y domain, papain-like domain, PPR, X domain, helicase and RNA dependent RNA polymerase). All possible ORFs were determined with ORF Finder.^2^ Each ORF was then submitted to BlastP versus the UniProtKB/SwissProt database^3^ to find corresponding sequences and identify ORF1, ORF2, and ORF3. ORF1 was aligned with the best Uniprot BlastP result and the matching domains were collected to create a GFF file which annotated each complete genome.

### Identification of Insertion or Duplication in the Hepatitis E Virus Genome

The annotated files were used to determine the length of each region in ORF1, ORF2, and ORF3 so as to identify strains with insertions. Analysis of the PPR took into account the fact that the length could vary from 183 to 315 nt, depending on the HEV clade. All sequences in each clade with longer than normal PPRs were considered to have insertions. The inserted segments were identified by aligning each complete genome sequence with the closest HEV sequence identified by BLAST on NCBI.^4^ The origin of the inserted segment (human or HEV genome) was then identified by a BLAST on NCBI. The duplicated regions were determined by aligning them on the complete genome with MUSCLE. The sequences of the recombinant variants have been deposited in the Genbank database under accession numbers MF444083, MF444086, MF444119, MF444145, MF444152, and MN646689-96 (Supplementary Table S1).

### Characterization of Insertions/Duplications

The sequences of all the PPRs were identified with reference to the 11,938 sequences of *Orthohepevirus A* (including 338 complete HEV genomes) available in the Virus Pathogen Resource (VIPR) database.^5^ Selected sequences were systematically searched to identify insertions so that they could be used, together with those identified by PacBio sequencing, for further analysis. The compositions of HEV PPR insertions/duplications were determined and their post-translational modifications predicted by analyzing a range of parameters. Potential ubiquitination sites were identified using the BDM-PUB server^6^ with a threshold of >0.3 average potential score. Potential phosphorylation sites were identified using the NetPhos 3.1 server^7^ with a threshold of >0.5 average potential score. Potential acetylation sites were identified using the Prediction of Acetylation on Internal Lysines (PAIL) server^8^ with a threshold of >0.2 average potential score. Potential N-linked glycosylation sites were identified using the NetNGlyc 1.0 server^9^ with a threshold of >0.5 average potential score. Potential methylation sites were identified using the BPB-PPMS server^10^ with a threshold of >0.5 average potential score. Nuclear export signal (NES) sites were identified using the Wregex server^11^ with parameters NES/CRM1 and Relaxed. Nuclear localization signal (NLS) sites were identified using SeqNLS^12^ with a 0.86 cut-off. The amino acid composition (proportions of amino acids), physico-chemical composition, and net load were analyzed with R. Principal component analysis (PCA) is a mathematical algorithm that reduces the dimensionality of the data while retaining most of the variation in a data set. PCA allows to identify new variables, the principal components, which are linear combinations of the original variables (Ringner, 2008). PCA was done (excluding the amino acid composition due to redundancy with physico-chemical properties) to summarize and visualize the information on the variables in our data set (Abdi and Williams, 2010); each variable was then studied independently. An in-house R-pipeline based on the amino acid sequences and the results of each analysis was used to generate bar plots for amino acid composition. The amino acid compositions were assigned to one of two categories: sequences with insertions/duplications (including insertions of human genome and HEV genome duplications) and sequences without insertions/duplications. The other parameters were assigned to one of three categories: sequences with insertions, those with duplications, and sequences without insertion/duplication.

## Results

### Characteristics of Hepatitis E Virus With Genomic Rearrangements

**Figure 1:**
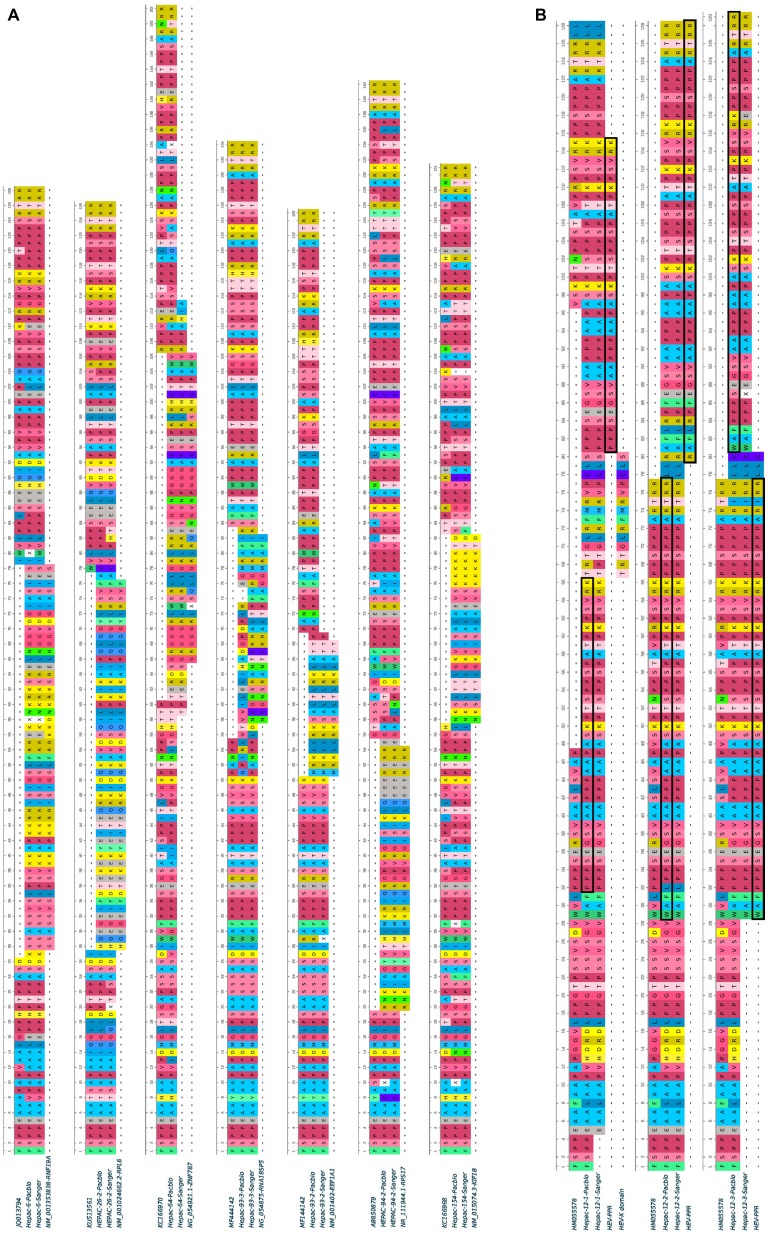
Human fragment insertions and duplications in the PPR of seven HEV GC sequences obtained by SMRT and Sanger sequencing. **(A)** Human fragment inserts. Variants 93-2 and 93-3 were characterized in the same patient. **(B)** HEV genome duplication and reference sequences. PPR duplications are boxed. Hyphen: gap.

Complete genome sequences were obtained for HEV strains from 114 HEV-infected patients. Most patients were sampled at the acute phase (92/114; 80%), of whom 29 were immunocompetent (29/92; 31.5%) and 63 were immunocompromised (63/92; 68.5%). The remaining 22 samples were taken from chronically infected immunocompromised patients during the chronic phase. Thus, 74.5% (85/114) of the samples were from immunocompromised patients: due to solid-organ transplantation (*n* = 61), hematological disease (*n* = 20), solid cancer (*n* = 2), or an immune disorder (*n* = 2). We found genomic rearrangements in the genomes of seven strains (7/114; 6.1%: 4 HEV-3f, 1 HEV-3e, 1 HEV-3 h, and 1 HEV-3chi-new). All the genomic rearrangements were found in immunocompromised patients (four solid organ transplant recipients and three patients with a hematological disease) (Table 1). Thus, the frequency of genomic rearrangements was 8.2% (7/85) in the immunocompromised patients. Three patients were acutely infected and four were chronically infected. All the genomic rearrangements were located in the PPR. The characteristics of each strain with genomic rearrangements are shown in Table 2. Virus-host recombinant variants were detected in six patients (Figure 1A and Supplementary Figure S2). Interestingly, one patient harbored two different recombinant variants (Hepac-93-2 and Hepac-93-3). Thus, seven recombinant host variants were identified in six patients. Another patient (Hepac-12) harbored three variants with duplications of HEV genes in the PPR (Figure 1B). The fragments of HEV genome were from the PPR + X domain (Hepac-12-1) or the PPR alone (Hepac-12-2 and Hepac-12-3) We found mixtures of variants with and without genomic rearrangements in the HEV from three patients infected for three months or less. We confirmed the sequences of all except one (Hepac-93-3, RNA18SP5) of these genomic rearrangement by Sanger sequencing (Figure 1). The discrepancy in Hepac-93-3 was due to a deletion of six nucleotides in the sequence obtained by SMRT.

**Table 1:** Characteristics of the patients infected by HEV-3 strain with genomic rearrangements.

HEV strain	Pathology of the patient	HEV diagnosis	Chronic/acute HEV infection at the time of diagnosis	Plasma HEV RNA concentration (log copies/ml)	Time between HEV infection and detection of genomic rearrangement
6	Chronic lymphoid leukemia	2014	Acute	7.5	<2 months
26	Renal transplant	2013	Acute	5.8	<2 months
94	Hepatic transplant	2013	Acute	6.6	<2 months
93	Hepatic transplant	2013	Chronic	7.5	3 months
154	Chronic lymphoid leukemia	2014	Chronic	5.3	5 months
64	Cardiac transplant	2009	Chronic	6.2	9 months
12	Light chain myeloma	2010	Chronic	7.7	10 months

**Table 2:** Characteristics of the naturally occurring genomic rearrangements in the PPRs of seven HEV genotype 3 strains.

HEV strain	HEV subtype	Nature of insertion	Number of variants	Name of the recombinant variant	Position in PPR (nt)	Nature of the inserted fragment	Percent of quasi species
6	3h	Human insert	1	Hepac-6	84–239	Ring finger protein 19A (RNF 19A)	100%
26	3chi-new	Human insert	2^*^	Hepac-26-2	81–230	Human ribosomal protein L6 (RPL6)	21%
94	3f	Human insert	2^*^	Hepac-94-2	57–164	Ribosomal protein 17S (RPS17)	63%
93	3f	Human insert	3^*^	Hepac-93-2	166–222	Eukaryotic translation elongation factor 1 alpha 1 pseudogene 13 (EEF1A1P13)	12%
				Hepac-93-3	164–242	18S ribosomal pseudogene 5 (RNA18SP5)	31%
154	3f	Human insert	1	Hepac-154	170–247	Kinesin family member 1B (KIF1B)	100%
64	3f	Human insert	1	Hepac-64	181–318	Zinc finger protein 787 (ZNF787)	100%
12	3e	HEV duplication	3	Hepac-12-1	93–239	PPR + X-domain	67%
				Hepac-12-2	85–237	PPR	22%
				Hepac-12-3	84–239	PPR	11%

* *Strains with one wild type variant*.

### Features of Polyproline Region With Insertion or Duplication

**Figure 2:**
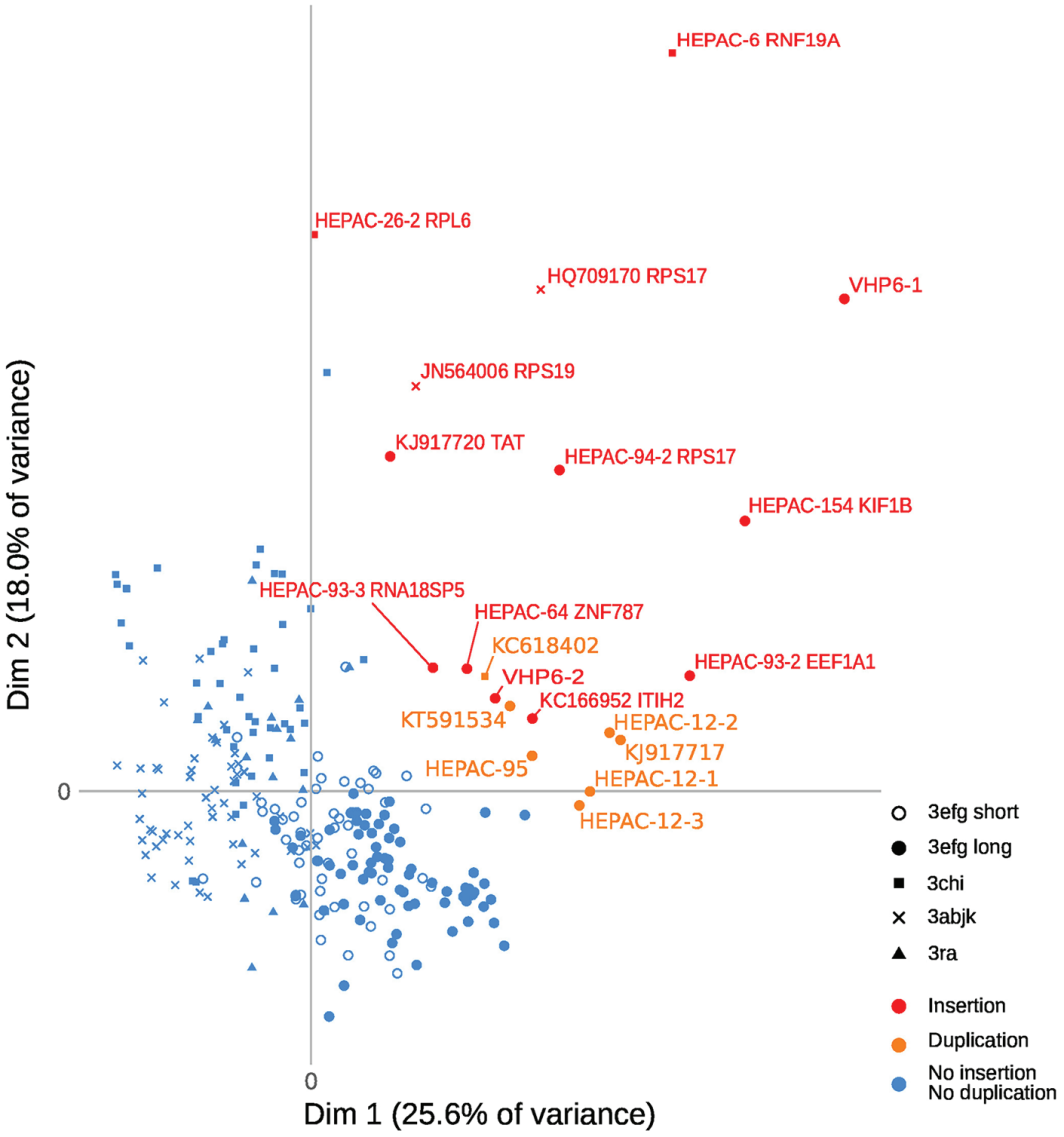
Principal Component Analysis of HEV-3 PPR sequences with insertions or duplications. Individual observations, each dot represents a sample. Each clade (3 abjk, 3 chi, 3 efg short or long, and 3ra) is represented by a symbol. The axes show the first two principal components [dimension 1 (dim 1) and dim 2], with the fraction of explained variance in parenthesis. Variables of the two components are detailed in Figure 3.

**Figure 3:**
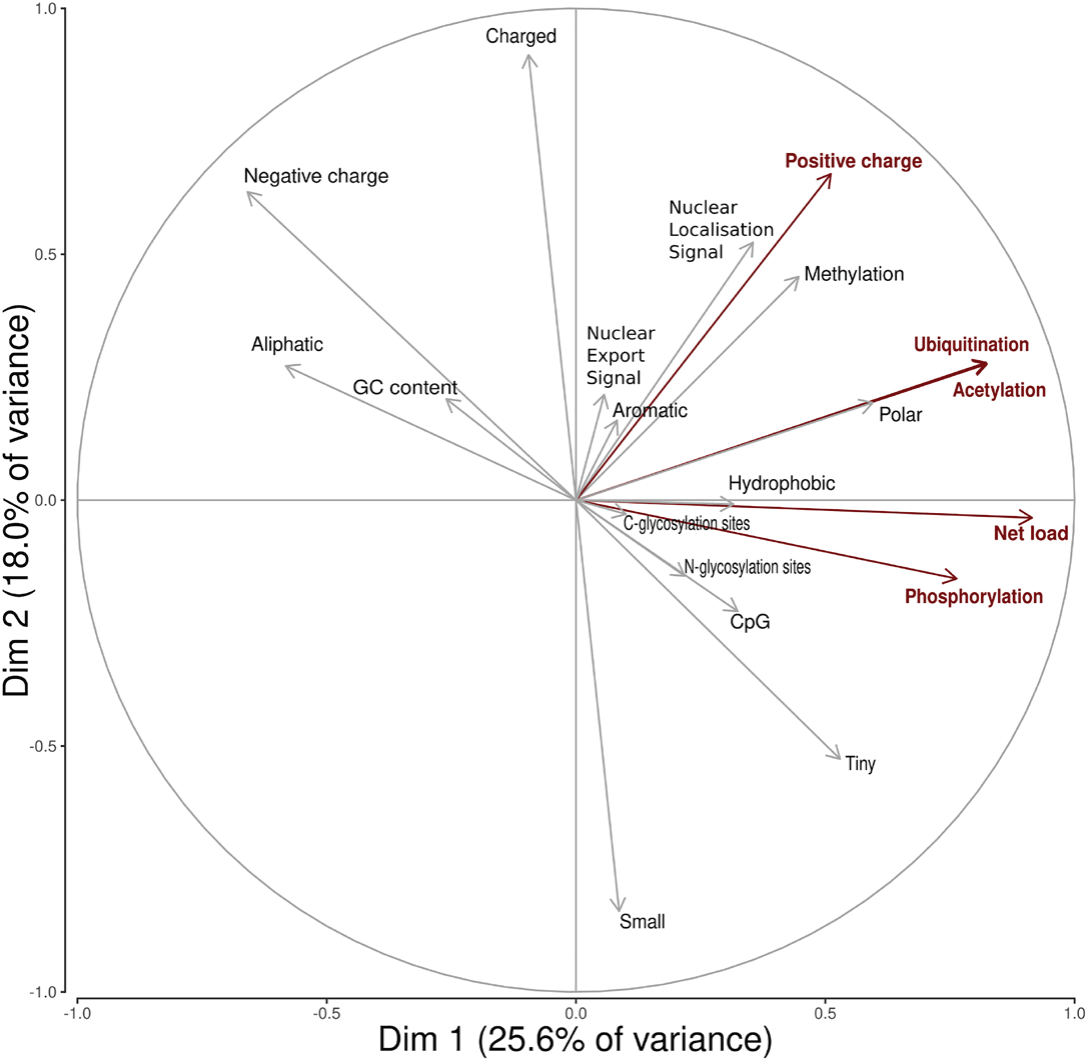
Principal Component Analysis variable circles of correlation. Variables characterizing insertions/duplications are shown in red (positive charge, net load, ubiquitination acetylation, phospohorylation sites). Dim1 is mainly composed of net load (16.4%), ubiquitination (13.3%), acetylation (13.2%), and phosphorylation (11.4%). Dim2 is mainly composed of positive charge (12.2%).

**Figure 4:**
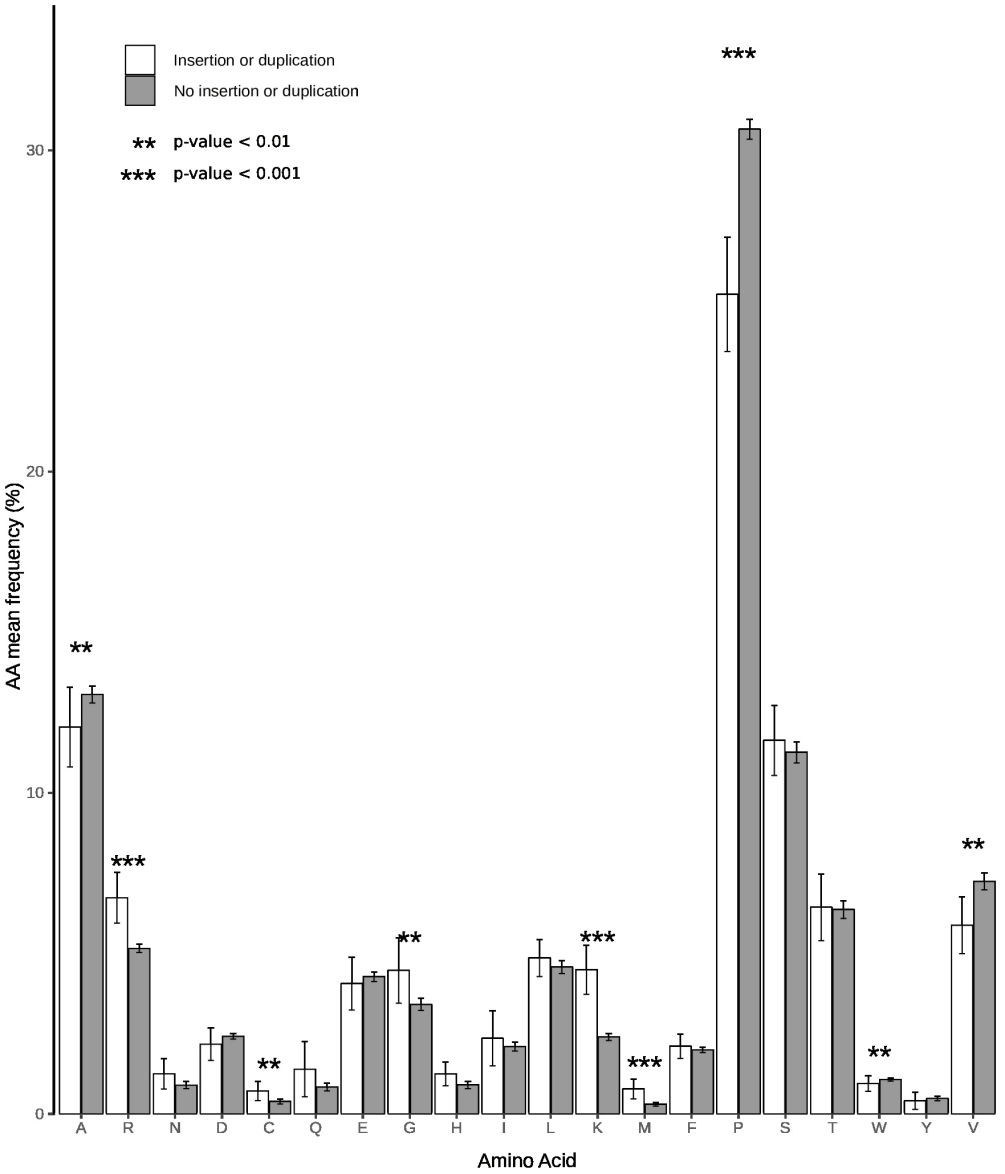
Amino acid compositions of PPR sequences with and without insertions/duplications. White bars represent sequences with insertion/duplication (*n* = 20) and black bars sequences without insertion/duplication (*n* = 294). Statistical differences between groups are indicated by stars. A *p* < 0.05 was considered significant.

A search in the VIPR database identified eight additional recombinant strains with genomic rearrangement in the PPR: HQ709170 (HEV-3a) with an RPS17 fragment (Shukla et al., 2011), strain JN564006 (HEV-3a) with an RPS19 fragment (Nguyen et al., 2012), strains KC166952, KJ917704, KJ917720 and KJ917717 (all HEV-3f) with an ITIH2 fragment, a PPR + RdRp duplication, a TAT fragment and a PPR duplication (Lhomme et al., 2014), strain KC618402 (HEV-3c) with a PPR duplication (Johne et al., 2014), and strain KT591534 (HEV-3f) with a PPR duplication not reported as a recombinant virus. Thus, we analyzed 13 PPR sequences with human gene fragment insertions and seven PPR sequences with duplications of HEV genome fragments (Supplementary Table S1). As all the genomic rearrangements occurred in HEV-3, analysis of strain with genomic rearrangements only included HEV-3 sequences (*n* = 294). Principal component analysis (PCA) is a mathematical algorithm that reduces the dimensionality of the data and allows to identify new variables, the principal components, which are linear combinations of the original variables. PCA was used to determine whether some variables in the data set were specific to the genomic rearrangements. The PCA separated sequences with genomic rearrangements from sequences without genomic rearrangements (Figure 2). The two principal components represented 43.6% of the variance. A detailed analysis of the components indicated that variables like the net load, positive charge, ubiquitination, acetylation, and phosphorylation seemed to be associated with sequences with genomic rearrangements (Figure 3). The features of sequences without genomic rearrangements, including HEV-3f short and long, did not differ with the length of the PPR. The amino acid composition encoded by genomes with and without genomic rearrangements is shown in Figure 4. Sequences with genomic rearrangements had increased Arg, Cys, Gly, Lys, and Met contents and decreased Ala, Pro, Trp, and Val contents. Human gene insertions encoded peptides rich in polar, positively charged amino acids (Arg, Asn, Gln, His, and Lys) and hydrophobic amino acid (Gly, Ile, and His) (Table 3). Insertions of HEV genome duplications encoded peptides rich in positively charged amino acids (Lys and Arg) and poor in negatively charged amino acid (Asp and Glu), while PPR sequences with genomic rearrangements had a significantly higher net load than sequences without genomic rearrangements (*p* < 0.001) (Table 3). The increased net load due to insertions resulted from increases in positively charged amino acids, whereas the increases caused by duplications were mainly due to fewer negatively charged amino acids. Sequences with genomic rearrangements had more ubiquitination (*p* < 0.001), acetylation (*p* < 0.001) and phosphorylation sites (*p* < 0.001) than did sequences without genomic rearrangements (Table 3), but there were no differences in methylation, N or O glycosylation sites.

**Table 3:** Impact of insertions on the amino acid composition, physico-chemical properties, and potential new regulation sites.

Variable	Sequences with human fragment insertions (*n* = 13)	Sequences with HEV genome duplication (*n* = 7)	Sequences without insertions/duplications (*n* = 294)	*p* (insertion/no insertion)	*p* (duplication/no duplication)
GC content (%)	48.5 (44.8; 51.9)	46 (45.3; 47.4)	46 (44; 49)	NS^±^	NS^±^
Small AA (%)	5.1 (2; 10.4)	5.5 (1.6; 12.8)	5.3 (1.9; 11.4)	NS^±^	NS^±^
Tiny AA (%)	8.3 (2; 10.7)	6.2 (1.7; 13.3)	6.7 (1.3; 12)	NS^±^	NS^±^
Positive charged AA (%)	4.2 (1.8; 7.1)	4.2 (0.82; 5.3)	2.7 (1.3; 4.5)	<0.01^±^	NS^±^
Negative charged AA (%)	3.2 (2; 4.5)	2.3 (1.6; 2.8)	2.9 (2.6; 4)	NS^±^	<0.01^±^
Charged AA (%)	3.8 (2; 5.7)	2.9 (1.5; 4.7)	2.7 (1.6; 4)	<0.01^±^	NS^±^
Aliphatic AA (%)	4.7 (3.6; 5.6)	4.2 (2.3; 5.5)	4 (2.7; 6.7)	NS^±^	NS^±^
Aromatic AA (%)	1 (0.75; 1.62)	0.8 (0.45; 1.6)	1.3 (0; 1.3)	NS^±^	NS^±^
Hydrophobic AA (%)	2.4 (1.8; 5.5)	2.4 (0.8; 4.7)	1.9 (1; 4.8)	0.04^±^	NS^±^
Polar AA (%)	2.4 (0.8; 6.5)	1.8 (0.8; 4.8)	2.5 (1; 4.8)	0.02^±^	NS^±^
Net load	5 (3; 6)	8 (5; 9)	0 (−1; 1)	<0.001^±^	<0.001^±^
Ubiquination sites	5 (3; 6)	6 (5.5; 6)	2 (1; 3)	<0.001^±^	<0.001^±^
Acetylation sites	5 (3; 6)	6 (5.5; 6)	2 (1; 3)	<0.001^±^	<0.001^±^
Phosphorylation sites	19 (16; 22)	19 (17; 19.5)	10 (8; 14)	<0.001^±^	<0.01^±^
Methylation sites	1 (0; 3)	0 (0; 0.5)	0 (0; 0)	<0.001^±^	NS^±^
Nuclear export signal sites (presence)	11 (84.6%)	7 (100%)	286 (97.3%)	<0.01^£^	NS^£^
Nuclear localization signal sites (presence)	3 (23.1%)	0 (0%)	1 (0.3%)	<0.001^£^	NS^¥^
N-Glycosylation (presence)	1 (7.7%)	0 (0%)	13 (4.4%)	NS^£^	NS^£^
C-Glycosylation (presence)	0 (0%)	0 (0%)	3 (1%)	NS^¥^	NS^¥^

*Data are numbers unless otherwise indicated. Variables are expressed as medians and interquartile ranges for Wilcoxon test, number (%) for chi2 or Fisher’s exact tests. AA: amino acids. NS: not significant. A *p* < 0.05 was considered significant*.

± *Wilcoxon test*.

¥ *Fisher’s exact test*.

£ *chi2 test*.

## Discussion

We generated and analyzed the near complete genome sequences of 114 HEV strains and found genomic rearrangements in 7/114 (6.1%). All the recombination detected were in the PPR of the HEV genomes from immunocompromised patients. The 10 inserted fragments were of a human gene or a duplication of part of the HEV genome. We detected recombinant virus/host variants at the acute phase of infection and found pure or mixed populations of variants with or without genomic rearrangements. We have found that these genomic rearrangements increase the net load of the PPR, with different mechanisms according to the nature of the inserted fragments: increase of positively charged amino acids in fragment from human genes and decrease of negatively charged amino acids in HEV gene duplication. Putative post-translation modifications were also found in recombinant variants.

We used SMRT PacBio sequencing to generate almost complete genome sequences. This third generation deep sequencing method can sequence single DNA molecules in real-time and generate long reads (Rhoads and Au, 2015). SMRT was used to identify genomic rearrangements because it enabled us to sequence longer fragments (up to 20 kb) than second-generation sequencing methods (< 500 bp).

All the genomic rearrangements obtained by SMRT sequencing except one were confirmed by Sanger sequencing, indicating that they are not artifacts. However, the fragment (RNA18SP5) inserted in one variant (Hepac-93-3) detected by SMRT was six nucleotides shorter than the sequence obtained by the Sanger method. This could be due to sequencing error not corrected by the bioinformatics pipeline, or it could reflect the presence of four variants: two detected by both methods and two others detected by either Sanger or SMRT sequencing.

All the new genomic rearrangements described herein were located in the HEV-3 PPR, as previously described by our group and others (Shukla et al., 2011; Nguyen et al., 2012; Johne et al., 2014; Lhomme et al., 2014). These recombinations were located at different positions in the PPR. Their presence in the PPR is not very surprising; the sequence encoding this region can vary in both composition and length depending of HEV clade and/or HEV subtype (Purdy et al., 2012; Lhomme et al., 2014). It is a region of great genetic flexibility: the PPR of HEV-3f viruses can be short (228 nt) or long (315 nt) due to a duplication of a PPR fragment (Purdy et al., 2012; Lhomme et al., 2014). A recent study also showed that HEV genomes harboring an epitope tag or NanoLuc in the PPR were found to be fully functional and allow for the production of infectious virus (Szkolnicka et al., 2019), confirming the remarkable flexibility of the PPR.

All the HEV genomic rearrangements described to date have been found in chronically infected patients (Shukla et al., 2011; Nguyen et al., 2012; Johne et al., 2014; Lhomme et al., 2014), but we have found genomic rearrangements at the acute phase in three HEV-infected patients. This raises the question of transmission of such recombinant variants at the acute phase. It is certainly more frequent in chronically HEV-infected patients; we reported a prevalence of 11% in chronically infected solid-organ transplant patients (Lhomme et al., 2014) and found that 8.2% of the immunocompromised patients in this study harbored recombinant variants. And four HEV strains had mixed populations of variants containing sequences with and without genomic rearrangements or different genomic rearrangements. Most of the mixed populations containing non-recombinant variants were found in patients infected for 3 months or less. Consequently, the time needed for recombinant variants to emerge still need to be clarified.

Several groups have shown that insertions of human fragments (RPS17, RPS19, ITI) (Shukla et al., 2011; Nguyen et al., 2012; Lhomme et al., 2014) give the virus a replicative advantage *in vitro* and that duplication helps it to adapt to cell culture systems (Johne et al., 2014). Although duplication of the virus genome has been found in several DNA viruses (Shackelton and Holmes, 2004), they appear to be infrequent in RNA viruses due to biological constraints, such as genome inflation (Simon-Loriere and Holmes, 2013). Duplications have been described in flaviviruses (Villordo et al., 2016), human respiratory syncytial virus (RSV) (Eshaghi et al., 2012; Schobel et al., 2016) and hepatitis C virus (HCV) (Le Guillou-Guillemette et al., 2015). Analysis of the RNA secondary structure of flavivirus 3’UTR revealed an association between RNA structure duplication and the ability of the virus to replicate in vertebrate and invertebrate hosts (Villordo et al., 2016). A 72-nucleotide duplication in the C-terminal region of the attachment glycoprotein gene of RSV genotype A was described (Eshaghi et al., 2012). As this glycoprotein is the target for neutralizing antibodies, such changes might alter the immunogenicity and pathogenicity of the virus. A duplication in the NS5A region of HCV has been described and may be associated with unfavorable evolution of the resulting liver disease, including possible involvement in liver carcinogenesis (Le Guillou-Guillemette et al., 2015). These strains with duplications were present in HCV genotype 1a and belonged to the same phylogenetic cluster. Several subtypes of HEV contain variants harboring duplications, although their impact on the pathophysiology of infection is still unknown. Duplications also occur in several RNA viruses but their locations differ: from the UTR, to structural and non-structural protein coding regions (Villordo et al., 2016), suggesting that they may influence virus function differently.

The present new, larger data set confirms earlier predictions that genomic rearrangements provide the PPR with putative new ubiquitination, acetylation, and phosphorylation sites (Lhomme et al., 2014). They also allow a higher net load. None of these features occurs in HEV-3f with a long PPR, suggesting that the differences are due to specific genomic rearrangements rather than PPR length. The fact that no new glycosylation or methylation sites were detected suggests that regulation sites are not acquired randomly. The peptides derived from the Kernow strain with reversed or reversed complementary insertions have fewer regulation sites, especially acetylation and ubiquitination sites, and they have no *in vitro* replicative advantage (Shukla et al., 2011). The conjugation of ubiquitin with a substrate usually leads to degradation of a peptide by the proteasome, and viruses, including HEV, can hijack the ubiquitin/proteasome system (UPS) (Karpe and Meng, 2012). The function of cellular enzyme can be modified by phosphorylation. Virus protein can also be phosphorylated (Jakubiec and Jupin, 2007): for example, phosphorylation of the hepatitis C virus NS5B has a regulatory role in HCV RNA replication (Kim et al., 2004, 2009; Han et al., 2014). Similarly, acetylation of histone and nonhistone proteins modulates protein function or the intracellular distribution of the acetylated protein (Sterner and Berger, 2000; Glozak et al., 2005). Acetylation of virus proteins can also modulate their function. Acetylation enhances the enzymatic activity of the HIV integrase and increases its affinity for DNA (Cereseto et al., 2005). More recently, it was shown that acetylation of highly conserved lysine residues might regulate specific functions of nucleoprotein in the viral life cycle of influenza A viruses, including viral replication (Giese et al., 2017). Lastly, we have found that the mechanisms by which human fragments and duplications increase the net load differ. Human fragment insertions increase the frequency of positively charged amino acids, while duplications seem to produce a small increase in positively charged amino acids and decrease the fraction of negatively charged amino acids. An increase in the net load in the V3 domain of the HIV glycoprotein 120 affects HIV tropism as the virus enters the host cell *via* the CXCR4 coreceptor rather than CCR5 (De Jong et al., 1992; Fouchier et al., 1992). The increase in the net load in the PPR of HEV could modify the way the virus proteins interact with host proteins. Although the lifecycle of HEV is not yet clear, we believe the PPR could regulate transcription and translation through ubiquitination, acetylation, or phosphorylation. These putative sites and their role must be confirmed by *in vitro* approaches.

In conclusion, we have described HEV strains with genomic rearrangements in patients at the acute phase of infection raising the possibility that such strains are directly transmitted. We have also shown that genomic rearrangements provide a higher net load with different features depending on the nature of the genomic rearrangement (duplication or insertion). Further studies are needed to clarify the role of these insertions/duplications by *in vitro* and conformational studies.

## Data Availability Statement

The sequences of the recombinant variants have been deposited in the Genbank database under accession numbers MF444083, MF444086, MF444119, MF444145, and MN646689-9.

## Ethics Statement

Biological materials and clinical data were obtained for a standard virus diagnosis, following physicians’ orders. This non-interventional study involved no additional procedures. Data were analyzed using an anonymized database. Such a protocol does not require written informed consent according to French Public Health law (CSP Art L 1121-1.1).

## Author Contributions

FN, SL, and JI designed the project, analyzed the results, and wrote the manuscript. NJ and FN performed the bio-informatics analyses. CD performed the biostatistical analysis. NK, J-MP, and LA provided the plasma samples. FA, CL, AR, MM, MD, and RC carried out the experiments. All the authors have approved the manuscript.

### Conflict of Interest

The authors declare that the research was conducted in the absence of any commercial or financial relationships that could be construed as a potential conflict of interest.

## Footnotes

^1^https://get.genotoul.fr/

^2^https://www.ncbi.nlm.nih.gov/orffinder/

^3^http://www.uniprot.org/

^4^https://www.ncbi.nlm.nih.gov/

^5^https://www.viprbrc.org

^6^http://bdmpub.biocuckoo.org/prediction.php

^7^www.cbs.dtu.dk/services/NetPhos/

^8^http://bdmpail.biocuckoo.org/prediction.php

^9^www.cbs.dtu.dk/services/NetNGlyc

^10^https://omictools.com/bpb-ppms-tool

^11^http://ehubio.ehu.eus/wregex/home.xhtml

^12^http://mleg.cse.sc.edu/seqNLS/index.html
