# Supplementary material for: Insertions and Duplications in the Polyproline Region of the Hepatitis E Virus
Source: Front Microbiol. 2020 Jan 31;11:1. doi: 10.3389/fmicb.2020.00001 (PMC7004952; doi:10.3389/fmicb.2020.00001)
Supplement: Supplementary file 1 [file Data_Sheet_1.docx]

Supplementary Material

# Supplementary tables

| **Supplementary Table 1:** HEV sequences with genomic rearrangements used to analyze features of inserted fragments. | | | | |
| --- | --- | --- | --- | --- |
| **Genbank number** | **Subtype** | **Genomic rearrangement** | **Inserted fragment** | **Reference** |
| HQ709170 | 3a | Insertion | RPS17 | Shukla, PNAS, 2011 |
| JN564006 | 3a | Insertion | RPS19 | Nguyen, J Gen Virol, 2012 |
| KC166952 | 3f | Insertion | ITIH2 | Lhomme, J Virol, 2014 |
| KJ917720 | 3f | Insertion | TAT | Lhomme, J Virol, 2014 |
| KJ917704 | 3f | duplication | PPR and RdRp | Lhomme, J Virol, 2014 |
| KJ917717 | 3f | duplication | PPR | Lhomme, J Virol, 2014 |
| KC618402 | 3c | duplication | PPR | Johne, J Viral Hepat, 2013 |
| KT591534 | 3f | duplication | PPR | unpublished |
| MF444145 | 3h | Insertion | RNF19A | Hepac-6 |
| MN646694 | 3chi-new | Insertion | RPL6 | Hepac-26-2 |
| MF444119 | 3f | Insertion | ZNF787 | Hepac-64 |
| MN646692 | 3f | Insertion | EEF1A1P13 | Hepac-93-2 |
| MN646695 | 3f | Insertion | RNA18SP5 | Hepac-93-3 |
| MN646693 | 3f | Insertion | RPS17 | Hepac-94-2 |
| MF444083 | 3f | Insertion | KIF1B | Hepac-154 |
| MF444086 | 3e | duplication | PPR and X-domain | Hepac-12-1 |
| MN646690 | 3e | duplication | PPR | Hepac-12-2 |
| MN646691 | 3e | duplication | PPR | Hepac-12-3 |
| MN646689 | 3f | Insertion | GATM | VHP-6-1 |
| MN646696 | 3f | Insertion | PEBP1 | VHP-6-2 |

# Supplementary figures


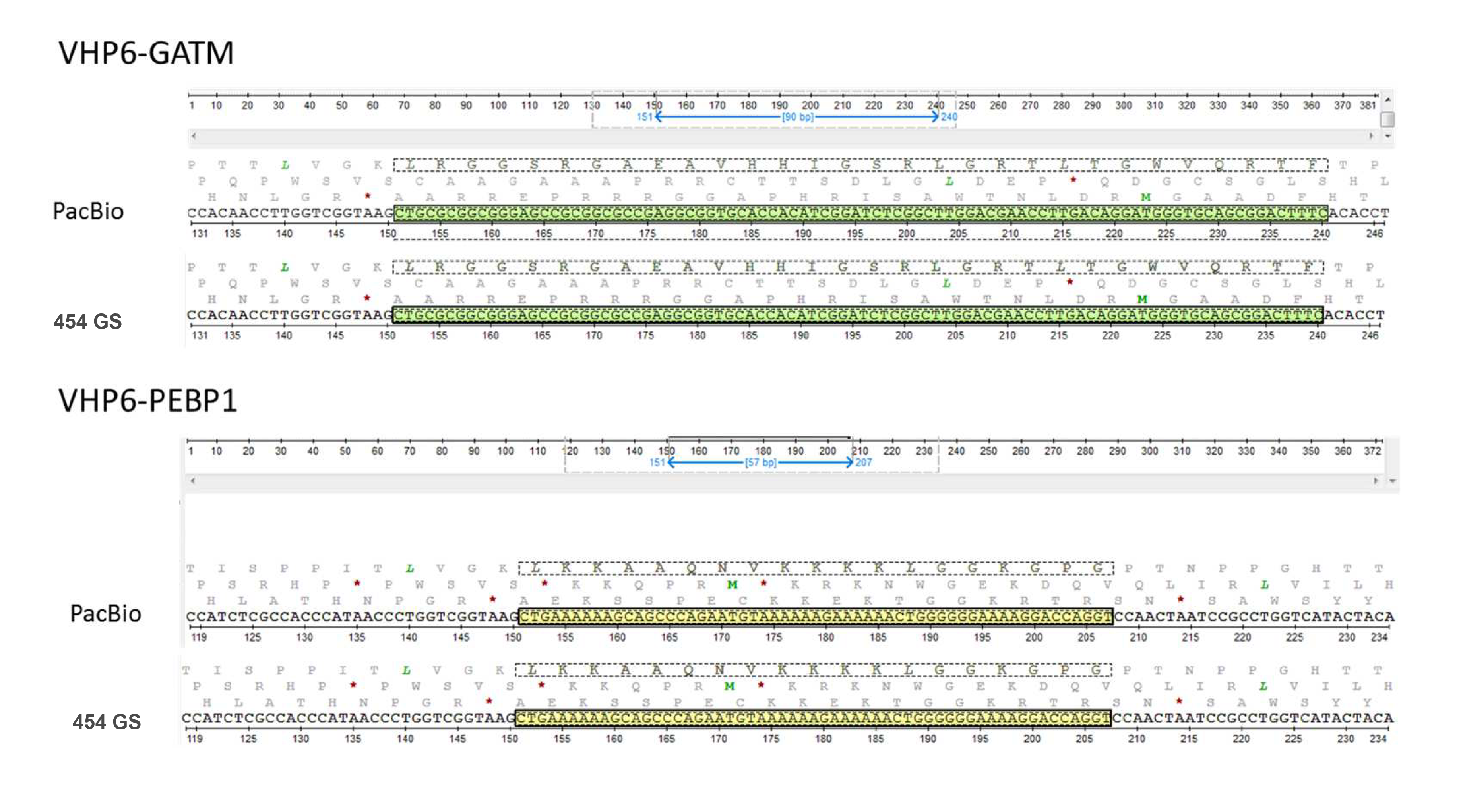


**Supplementary figure 1:** Human genome fragment insert in the VHP6 strain identified by SMRT sequencing and shot-gun pyrosequencing (454 GS Junior system). There are two variants: one with a fragment of human GATM and the other with a fragment of human PEBP1.


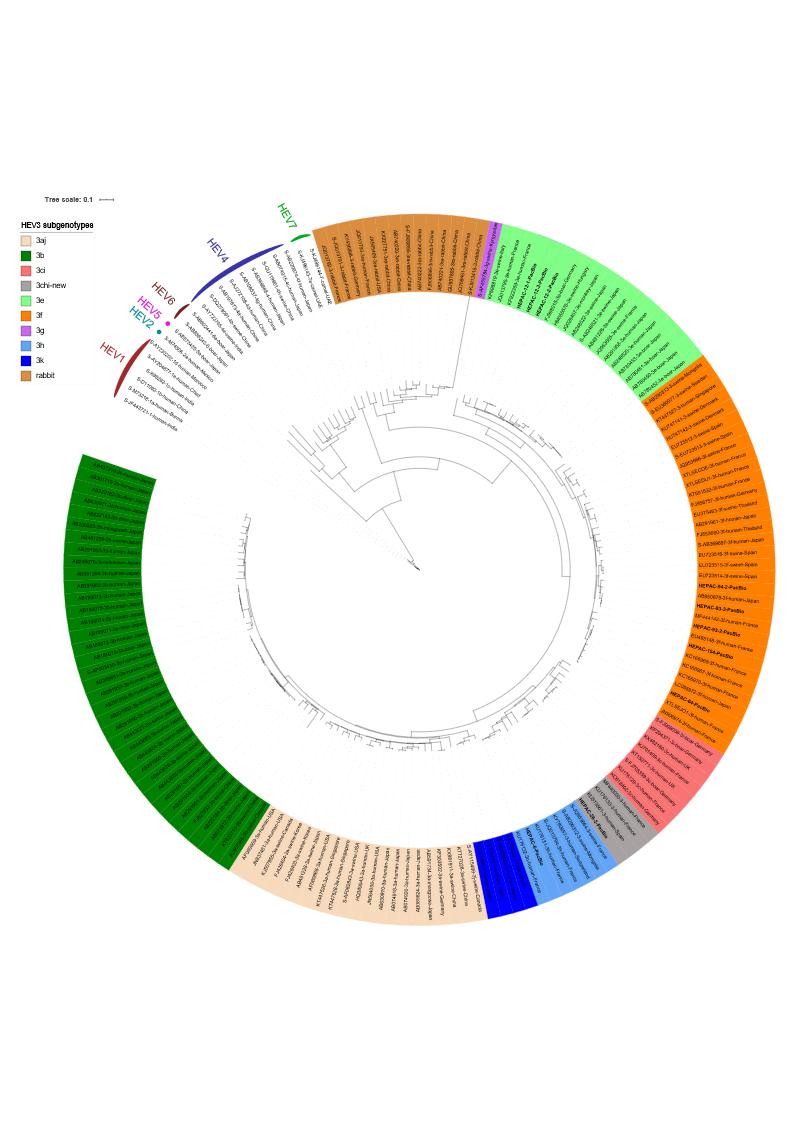


**Supplementary figure 2:** Phylogenetic analysis of complete hepatitis E virus sequences. Complete hepatitis E virus genomes of ~7,250 nucleotides were used to make the phylogenetic tree. Phylogenetic maximum likelihood tree automated partition defining subtypes. The Phylogenetic tree was visualized with iTOL. Subgenotypes are color labelled and variants discussed in this study are written in bold.
